# A preliminary validation of the Brief COPE instrument for assessing coping strategies among people living with HIV in China

**DOI:** 10.1186/s40249-015-0074-9

**Published:** 2015-09-14

**Authors:** Xiao-you Su, Joseph TF Lau, Winnie WS Mak, KC Choi, Tie-jian Feng, Xi Chen, Chu-liang Liu, Jun Liu, De Liu, Lin Chen, Jun-min Song, Yan Zhang, Guang-lu Zhao, Zhang-ping Zhu, Jin-quan Cheng

**Affiliations:** Chinese Academy of Medical Sciences & Peking Union Medical College, Beijing, China; JC School of Public Health and Primary Care, The Chinese University of Hong Kong; CUHK Shenzhen Research Institute, Shenzhen, China; Department of Psychology, The Chinese University of Hong Kong, Hong Kong, China; Shenzhen Chronic Disease Hospital, Shenzhen, China; Hunan Province CDC, Hengyang, China; Hengyang City CDC, Hengyang, Hunan Province China; The 5th Hospital, Hengyang, Hunan Province China; Shenzhen CDC, Shenzhen, China; The 3rd Hospital, Hengyang, Hunan Province China

**Keywords:** People living with HIV, Brief COPE, Confirmatory factor analysis, Explanatory factor analysis, Perceived Social Support Scale, Perceived Discrimination Scale for PLWH, China

## Abstract

**Background:**

The Brief COPE instrument has been utilized to conduct research on various populations, including people living with HIV (PLWH). However, the questionnaire constructs when applied to PLWH have not been subjected to thorough factor validation.

**Methods:**

A total of 258 PLWH were recruited from two provinces of China. They answered questions involving the scales of three instruments: the Brief COPE, the Perceived Social Support Scale, and the Perceived Discrimination Scale for PLWH. Confirmatory factor analysis (CFA) and exploratory factor analysis (EFA) were conducted.

**Results:**

The CFA found a poor goodness of fit to the data. The subsequent EFA identified six preliminary factors, forming subscales with Cronbach’s alphas, which ranged from 0.61 to 0.80. Significant correlation coefficients between the subscales and measures of perceived social support and perceived discrimination were reported, giving preliminary support to the validity of the new empirical factor structure.

**Conclusion:**

This study showed that the original factor structure of the Brief COPE instrument, when applied to PLWH in China, did not fit the data. Thus, the Brief COPE should be applied to various populations and cultures with caution. The new factor structure established by the EFA is only preliminary and requires further validation.

**Electronic supplementary material:**

The online version of this article (doi:10.1186/s40249-015-0074-9) contains supplementary material, which is available to authorized users.

## Multilingual abstracts

Please see Additional file 1 for translations of the abstract into the six official working languages of the United Nations.

## Background

Coping is defined as constantly changing cognitive and behavioral efforts to manage specific external and/or internal demands that are taxing or exceeding the resources of a person [1]. It may significantly amplify or diminish the effects of stress or adverse events [2] as different types of coping strategies can have protective or harmful effects on individuals’ health and wellbeing [3–5].

People living with HIV (PLWH) face severe challenges such as stigma [6, 7], poor antiretroviral adherence [8], various stresses [9], and depression [10, 11]. Thus, there is a strong need to identify protective and risk factors as well as effective coping strategies associated with mental health problems, in order to develop evidence-based prevention programs targeting PLWH [12]. Research on PLWH found that appropriate coping strategies are associated with better mental health and quality of life [13], decreased depressive symptoms [14], effective stress management, and better drug adherence [15]. Furthermore, elaborating on the structure of coping strategies of PLWH would also help in facilitating and evaluating the community-based interventions that have been proved effective in averting risk behaviors and disease burden of infectious diseases in developing countries [16, 17].

The COPE Inventory was developed to assess a broad range of coping responses. It’s based on the coping model developed by Lazarus and Folkman [1], and the behavioral self-regulation model developed by Carver and Scheier [18, 19]. It has 60 items, consisting of 15 subscales (four items per scale), each with a specific conceptual focus. Good psychometric properties including high values of Cronbach’s alpha, test-retest reliability, and significant correlations with external variables have been reported [20]. There is no fully validated Chinese version of the COPE Inventory.

The Brief COPE [21] is the abbreviated version of the COPE Inventory and assesses dispositional as well as situational coping efforts [20]. The 28-item Brief COPE (consisting of 14 subscales) has acceptable psychometric properties and has been used extensively to examine the relationship between various coping strategies and psychological outcomes in PLWH [22–24] and other populations [25]. Its constructs, however, have not been subjected to factor validation. Although the Chinese version of the Brief COPE was translated into Chinese and back-translated into English, a test for internal consistency and factor analysis has not yet been performed [26].

The estimated number of PLWH in China was over 1,000,000 in 2014. Few psychological support services exist despite the highly stressful and discriminating social environment [27]. The investigation of coping responses to stressors within the cultural context is critically important. There is, however, a dearth of such data in China, possibly due to the lack of fully validated Chinese instruments assessing coping strategies. PLWH in different countries may use coping strategies differently. Prior studies advocated a culturally sensitive approach to ensure that the impact of interventions is optimized to benefit the individuals recovering from a stressful event [28]. In addition to social prejudices, Chinese PLWH encounter difficulties due to HIV/AIDS-related symptoms and complications, low socioeconomic status, a conservative social environment [27], and a lack of social support [29].

## Methods

### Overall study design

**Fig. 1:**
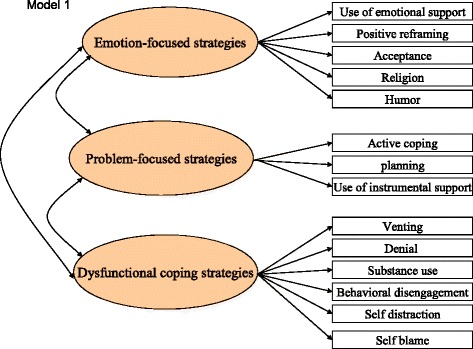
Three-category model of Brief COPE developed by Cooper et al

**Fig. 2:**
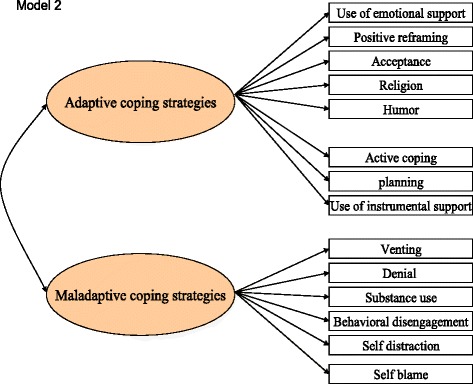
Two-category model of Brief COPE developed by Meyer et al

This study investigated the psychometric properties of the Chinese version of the Brief COPE among PLWH in China. The original Brief COPE has 14 subscales (self-distraction, active coping, denial, substance use, use of emotional support, use of instrumental support, behavioral disengagement, venting, positive reframing, planning, humor, acceptance, religion, self-blame) comprising two subscales each. Two previously specified second order factor models of the Brief COPE were tested using the confirmatory factor analysis (CFA). The two measurement models [25, 30] are presented in Figs. 1 and 2. In Fig. 1, the first model grouped the 14 subscales of the Brief COPE into three categories: problem-focused (active coping, planning, use of instrumental support), emotion-focused (use of emotional support, positive reframing, acceptance, religion, humor), and dysfunctional coping (venting, denial, substance use, behavioral disengagement, self-distraction, self-blame) [25, 31]. Dysfunctional coping correlated with depressive symptoms, whereas mixed findings were reported on the relationship between problem-focused and emotion-focused coping strategies and psychological outcomes [25, 30].

In Fig. 2, the second model grouped the 14 subscales under adaptive coping (including all the subscales of the problem-focused and emotion-focused coping categories in the first model) and maladaptive coping strategies (including all the subscales of dysfunctional coping in the first model) [30]. Adaptive coping strategies tend to be associated with desirable outcomes and maladaptive coping strategies tend to be associated with undesirable outcomes [30, 32]. Previous studies on Brief COPE have used either the combined subscales (e.g., adaptive *versus* maladaptive coping) [25, 30, 33] or the separate subscales [26, 34–36].

In this study, we performed CFA based on the two aforementioned models. In cases where the CFA model showed a poor goodness of fit to the data, exploratory factor analysis (EFA) was performed, in order to establish the factor structure of the Brief COPE. We hypothesized that perceived social support would be positively correlated with subscale scores related to active coping strategies [37], whereas perceived discrimination would be associated with scores related to maladaptive coping strategies [38, 39].

### Procedure and participants

The study population included Chinese PLWH aged between 18 and 60 years, who had known their confirmed positive HIV status for at least three months. From September 2007 to January 2008, a total of 258 PLWH were recruited from Hengyang city, Hunan province and Shenzhen city, Guangdong province, in China. The participants were recruited from Centers for Disease Control and Prevention (CDCs), hospital HIV clinics, and methadone maintenance treatment (MMT) clinics. Participants were interviewed once identified by multiple sources (CDCs, HIV clinics and MMT clinics). PLWH were contacted *via* phone or invited to participate while they were using HIV-related services (such as having a CD4 test, attending a MMT clinic, getting counseling from the hospital/CDCs, or when receiving prescribed drugs from the hospital). The overall response rates, defined as the number of participants who completed the questionnaire divided by the number of participants who were invited to participate, were 71.4 % in Shenzhen and 75.4 % in Hengyang. Breakdowns of the response rates are shown in Table 1.

**Table 1:** Recruiting procedure and response rates in Hengyang and Shenzhen city

		Invited by phone (C/I)	Invited when using services (C/I)	Total (C/I)	Response rate
Hengyang	CDC	40/54	15/22		
	HIV clinic	60/85	50/60	193/256	75.40%
	MMT clinic	N/A	28/35		
Shenzhen	CDC	40/60	25/31	65/91	71.40%

C/I: numbers completed questionnaires/numbers being invited

Participants were briefed about the purpose and the confidential nature of the study. After written informed consent was obtained, they were interviewed face-to-face in a private room at their local CDC or at the hospitals/clinics. The Hengyang participants were interviewed by the first author and two other trained doctors from the CDC, whereas the Shenzhen participants were interviewed by the first author and one of the co-authors (Song). No personal information was recorded in the questionnaire and a monetary incentive of 50 Yuan (US$ 6.25) was offered to the participants for their time. Approval was obtained from the ethics committee of the Chinese University of Hong Kong.

### Measures

#### Demographic characteristics

Information was collected on the participants’ demographic characteristics (age, gender, educational level, marital status, income, employment). Participants were also asked about their perceived mode of HIV transmission, antiretroviral treatment status, and length of time since HIV diagnosis.

#### Brief COPE

The strategies adopted by PLWH to cope with stress in the previous three months were assessed using the 28-item Brief COPE, comprising 14 two-item subscales. The Cronbach’s alpha for the original subscales ranged from 0.50 (venting) to 0.90 (substance use) [21]. The individual item score of the Chinese Brief COPE [26] ranged from 1 (not doing it at all) to 4 (doing it a lot).

#### The Perceived Social Support Scale (PSSS) questionnaire

The PSSS is a validated 12-item questionnaire that assesses the perceived support from family, friends, and significant others [40]. The scores range from 12 to 84, with a higher score indicating a higher level of perceived support. In a previous study, the Chinese version of the PSSS was validated and showed good internal reliability (Cronbach’s alpha = 0.89) [41]. In this study, the Cronbach’s alpha was also 0.89.

#### Perceived Discrimination Scale for PLWH (PDSHIV)

This scale was constructed by the authors and was used in other published papers [10, 42]. Based on a literature review [43, 44], six items were generated: “Family members are unwilling to live with PLWH”; “Healthcare workers refuse to provide PLWH services or provide lower-quality services”; “Friends are reluctant to be affiliated with a HIV infected person”; “PLWH are fired once the employer knows of their HIV status”; “Most people would discriminate against PLWH”; and “Family members of PLWH are being looked down upon by their relatives and neighbors.” Item responses included: “not true at all” (scored 0), “hardly true” (scored 1), “somewhat true” (scored 2), and “completely true” (scored 3). A higher score indicates a higher level of perceived discrimination. The EFA extracted a one-factor solution from the six items, which explained 41.9 % of the total variance. The CFA on the one-factor solution of PDSHIV also showed an acceptable model fit (RMSEA = 0.08, NFI = 0.98, CFI = 0.99, GFI = 0.99). In this study, the composite scale had an alpha value of 0.70.

### Statistical analysis

Descriptive statistics were presented. Cronbach’s alphas were derived to assess internal validity of the subscales. The item responses in the two models of Brief COPE (shown in Figs. 1 and 2) were specified and estimated using the diagonally weighted least squares chi-square utilizing the LISREL 8.50. Relative chi-square (*χ*2) was calculated (weighted least squares chi-square divided by degrees of freedom) to indicate the model fit. The appropriateness of each latent construct was evaluated in terms of the comparative fit index (CFI), the root mean square error of approximation (RMSEA), the normed fit index (NFI), the non-normed fit index (NNFI), and the goodness of fit index (GFI). An acceptable relative *χ*2 fit index is usually set at a 3:1 ratio, whereas some researchers consider a higher ratio of around 5:1 [45]. Values of CFI, GFI, NFI, and NNFI that were 0.90 or greater [45] and RMSEA values of 0.08 or less [46] were indicative of a good model fit, whereas RMSEA values of 0.05 or less were considered to be a near-perfect fit [47].

In the EFA, a varimax rotation was performed to yield a certain number of factors; the number of factors was determined by eigenvalues [48] and factor loadings [49, 50]. The Kaiser-Meyer-Olkin (KMO) and Bartlett’s sphericity tests were used to measure sampling adequacy. Subscale scores were derived by adding the item scores of individual factors. Item-subscale correlation coefficients were derived and Cronbach’s alpha coefficients [51] were estimated. Cronbach’s alpha, a measure of the average correlation of items within a scale, is a commonly used statistic to assess internal consistency. A commonly accepted standard for internal consistency is 0.60 [52]. Spearman correlation coefficients among different subscale scores of the Brief COPE and PSSS (measure of social support)/PDSHIV (measure of perceived discrimination) were derived. The content validity of the factors was evaluated by a panel including one psychologist, one epidemiologist, and one PhD student in Social Medicine, who were all experienced in HIV/AIDS research. The panel members made their judgments on the relevance of the items and ensured that the formulation of the specific subscales had clear meanings.

## Results

### Demographic characteristics of the respondents

Of the respondents, 73.6 % were male; 53.1 % were 30 to 39 years old; 41.5 % were married; 63.6 % had attained junior high or below education; and 47.7 % had no income. Respectively, 29.6, 58.3, and 12.2 % reported that they were infected *via* heterosexual, intravenous drug use, and men who have sex with men behavior. Of all the participants, 43.8 % were receiving antiretroviral treatment and 26.7 % were diagnosed with HIV before 2005 (see Table 2).

**Table 2:** Background characteristics of the respondents (n = 258)

		% (n=258)
Demographic variables		
Gender	Male	73.6
	Female	26.4
Age (years)	29 or below	22.9
	30–39	53.1
	40 or above	24
Local resident	Yes	72.9
	No	27.1
Marital status	Currently married	41.5
	Currently not married	58.5
Whether having kid(s)	Yes	47.3
	No	52.7
SES variables		
Education level	Primary or below	12
	Junior high	51.6
	Senior high/technical secondary school	27.5
	Junior College or above	8.9
Employment status	Employed	25.2
	Unemployed	67.1
Individual monthly income	No income	47.7
	<=500 yuan	15.5
	500 to 1000 yuan	10.5
	1000 to 2000 yuan	11.2
	>2000 yuan/month	12.8
Whether have health insurance	Yes	21.7
	No	77.5
HIV related variables		
Probable mode of HIV transmission	MSM behavior	12.2
	Heterosexual behavior	29.6
	IDU	58.3
Currently receiving ARV therapy	Yes	43.8
	No	56.2
Year confirmation of HIV status	1997 to 2005	26.7
	2006 to 2008	72.5

### Results of the CFA based on the previous two models

Relevant statistics are summarized in Table 3. With respect to Model 1 and Model 2, the relative *χ*2 index, the test of absolute model fit, was respectively 6.81 and 6.60 (*df* = 350, 350, *p* < 0.05), showing a poor absolute fit between the two models and the data. The comparative model fit (represented by RMSEA, NFI, and NNFI), the CFI, and the GFI were also poor (0.15, 0.71, and 0.72, respectively).

**Table 3:** Indices of CFA for Model 1 and Model 2

Model	No. of factors	χ^2^(d.f.)	Relative χ^2^ fit index	RMSEA	NFI	NNFI	CFI	GFI
Model 1	3	2382.16 (350)	6.81	0.15	0.71	0.72	0.74	0.60
Model 2	2	2315.62 (351)	6.60	0.15	0.71	0.72	0.74	0.61

*RMSEA* root mean square of approximation, *NFI* normed fit index, *NNFI* non-normed fit index, *CFI* comparative fit index, *GFI* goodness of fit index

Model 3: model generated by EFA in this study

**Table 4:** Factor loadings of the Brief COPE

	Factors					
Items of Brief COPE	1	2	3	4	5	6
14. I've been trying to come up with a strategy about what to do	**0.70**	−0.06	0.12	0.29	0.04	0.01
12. I've been trying to see it in a different light, to make it seem more positive	**0.68**	0.10	0.13	−0.12	−0.01	0.24
25. I've been thinking hard about what steps to take	**0.66**	0.04	0.25	0.10	0.02	0.11
2. I've been concentrating my efforts on doing something about the situation I'm in	**0.66**	−0.02	0.04	0.13	0.07	0.10
7. I've been taking action to try to make the situation better	**0.65**	0.13	0.01	0.13	0.01	0.10
17. I've been looking for something good in what is happening	**0.60**	−0.10	0.23	−0.01	0.27	0.04
24. I've been learning to live with it	**0.53**	0.02	0.20	0.00	0.19	0.21
20. I've been accepting the reality of the fact that it has happened	**0.42**	0.29	0.03	−0.37	0.39	−0.22
11. I've been using alcohol or other drugs to help me get through it	−0.09	**0.88**	0.07	0.07	0.05	0.04
4. I've been using alcohol or other drugs to make myself feel better	−0.01	**0.68**	0.09	0.10	0.01	−0.02
16. I've been giving up the attempt to cope	0.11	**0.55**	0.21	0.22	0.08	0.08
21. I've been expressing my negative feelings	0.15	**0.50**	0.09	0.17	0.16	0.33
6. I've been giving up trying to deal with it	0.09	**0.40**	−0.00	0.28	0.25	0.35
10. I’ve been getting help and advice from other people	0.22	0.11	**0.77**	0.08	0.06	−0.12
23. I’ve been trying to get advice or help from other people about what to do	0.21	0.12	**0.76**	−0.05	0.23	−0.01
15. I've been getting comfort and understanding from someone	0.12	−0.01	**0.67**	−0.05	0.08	0.32
5. I've been getting emotional support from others	0.01	0.12	**0.64**	0.09	−0.05	0.31
9. I've been saying things to let my unpleasant feelings escape	0.38	0.26	**0.54**	0.22	0.10	−0.03
3. I've been saying to myself "this isn't real."	0.11	0.11	−0.09	**0.72**	0.02	0.18
8. I've been refusing to believe that it has happened	0.23	0.17	0.08	**0.71**	−0.04	0.04
26. I’ve been blaming myself for things that happened	0.03	.12	0.11	**0.68**	0.41	−0.07
13. I’ve been criticizing myself	0.09	0.29	0.13	**0.61**	0.36	−0.06
22. I've been trying to find comfort in my religion or spiritual beliefs	0.13	0.12	0.12	0.12	**0.78**	0.19
27. I've been praying or meditating	0.10	0.07	0.14	0.38	**0.66**	0.22
19. I've been doing something to think about it less, such as going to movies, watching TV, reading, daydreaming, sleeping, or shopping	0.21	−0.06	0.15	0.04	0.05	**0.67**
28. I've been making fun of the situation	0.16	0.27	0.08	0.07	0.31	**0.48**
18. I've been making jokes about it	0.30	0.20	0.22	−0.12	0.29	**0.46**
1. I've been turning to work or other activities to take my mind off things	**0.45**	0.17	−0.04	0.23	−0.01	**0.45**
Cronbach’s alpha	0.80	0.76	0.78	0.76	0.76	0.61
Item-subscale correlation coefficients	0.31 − 0.58	0.42 − 0.66	0.48 − 0.64	0.54 − 0.59	0.62	0.31 − 0.46
Variance explained	13.07 %	9.81 %	9.75 %	9.28 %	7.01 %	6.61 %

Bold numbers in each column indicates the items with similar factor loadings

### Results of the exploratory factor analysis

Due to the poor fit between the two models and the data, an EFA were conducted to establish the factor structure of the 28-item Brief COPE. The KMO value was 0.84 meeting the criterion of KMO > = 0.60. The Bartlett’s sphericity test was statistically significant (*χ*^2^ = 2572, *df* = 378, *p* < 0.000). The varimax rotation yielded six factors using the criterion of eigenvalue larger than 1, explaining 56.1 % of the total variance. The minimum item loading was 0.40 and there was only one item with cross-loadings >0.40 (“I’ve been turning to work or other activities to take my mind off things.”) This item was assigned to one of the two factors that allowed for better interpretation (see Table 4).

The panel members discussed the specific subscales in terms of their meanings to ensure content validity. The first factor consisted of four subscales of the original Brief COPE (positive reframing, planning, active coping, and acceptance) and was named “Problem-solving and acceptance.” The second factor consisted of five items (substance use and behavioral disengagement subscales, and one item from the venting subscale) and was named “Negative venting and avoidance.” The third factor consisted of five items (use of instrumental support and use of emotional support subscales, and one item from venting subscale) and was named “Support seeking.” The fourth factor consisted of four items (denial and self-blame subscales) and was named “Self-blame and denial.” The fifth factor only consisted of the two items of the religion subscale and was named “Reliance on spirituality.” The sixth factor consisted of four items of the humor and self-distraction subscales, and was named as “Humor and self-distraction.” The double-loaded item (“I’ve been turning to work or other activities to take my mind off things”) was assigned to the sixth factor, as its meaning was consistent with the interpretation of this factor. The means (SD) of the subscales related to the six factors are shown in Table 5.

**Table 5:** Correlation coefficients between the six subscales and PSSS/PDSHIV

	Mean (std)	PSSS	PDSHIV
Problem-solving and acceptance	18.01 (4.39)	0.24^**^	−0.08
Negative Venting and avoidance	8.69 (2.80)	−0.03	0.11
Support seeking	8.81 (2.78)	0.30^**^	−0.12^*^
Self-blame and denial	8.64 (2.87)	−0.12	0.11
Reliance on spirituality	3.07 (1.35)	0.02	0.04
Humor and self-distraction	8.26 (2.23)	0.17^**^	−0.06

*PSSS* The Perceived Social Support Scal*e*, *PDSHIV* Perceived Discrimination Scale for PLWH

*p<0.05; **p<0.01

### Inter-item correlations and internal consistency of the subscales that were identified from the EFA

The item-total spearman correlation coefficients of the six factors ranged, respectively, from 0.31 to 0.58, 0.42 to 0.66, 0.48 to 0.64, 0.54 to 0.59, 0.62, and 0.31 to 0.46. These figures were found to be statistically significant (*p* < 0.05) and acceptable [53, 54]. The Cronbach’s alpha coefficients of the six subscales ranged from 0.61 to 0.80 (see Table 5), which were also found to be acceptable according to our pre-set criterion of 0.60 [52].

### Construct validity of the structure identified from the EFA

Table 5 shows that the “Support seeking” subscale was positively correlated with the PSSS (r = 0.30, *p* < 0.05) and negatively correlated with the PDSHIV (r = −0.12, *p* < 0.05). The “Problem-solving and acceptance” subscale was positively correlated with the PSSS (r = 0.24, *p* < 0.05). The “Humor and self-distraction” subscale was positively correlated with the PSSS (r = 0.17, *p* < 0.05).

## Discussion

In recent years, the literature on coping with stress related to chronic diseases, life-threatening illnesses, and natural disasters has grown substantially [55–57]. Coping strategies adopted by PLWH have very important practical applications as such strategies determine health outcomes and even chances of spreading HIV to others [13]. Amongst PLWH, coping strategies such as behavioral disengagement and denial are found to be associated with disease progression and a poor psychological status, whereas the coping strategy of acceptance is associated with a lower level of distress [58, 59]. There are, however, mixed findings about the effect of higher-order coping strategies (e.g., problem-focused and emotion-focused coping) on psychosocial outcomes [25, 31–33].

The COPE Inventory and the Brief COPE have been used in a number of HIV-related studies [34, 60]. The Brief COPE has sometimes been used in the absence of full validation, which may be problematic. The most important finding of this study was that the CFA results did not fit the data. The results hence neither supported the grouping of the subscales into the emotion-focus, problem-focus, or dysfunctional coping strategies, nor into the adaptive and maladaptive coping strategies [25, 30]. Consistently, the author of COPE and Brief COPE did not recommend combining related subscales into “problem-focus” and “emotion-focus” indices, or to form an “overall” index. Instead, he suggested that researchers use separate subscales or the factors obtained from EFA when investigating associations between coping and other variables [21]. Such findings and notions should be taken into account when using the Brief COPE instrument.

Our subsequent EFA generated six preliminary factors. The psychometric properties of the new factor structure were found to be acceptable (*e.g.,* KMO, Bartlett’s sphericity test, and Cronbach’s alphas of the factors). However, we acknowledge that the naming of the subscales was subjective although a panel was involved. We contend that such preliminary factors were consistent with the cultural context and the literature. For instance, two of the six generated factors (“Humor and self-distraction” and “Problem-solving and acceptance”) potentially reflected the resilient nature of the traditional Chinese culture. As some popular ancient Chinese proverbs, such as “Ku zhong qiu le” (苦中求乐, “pursue happiness while suffering”) and “Sui yu er an” (随遇而安, “make the best out of one’s circumstances”), indicate Chinese culture is highly resilient to cope with harsh conditions. Furthermore, the two factors of “Negative venting and avoidance” (such as use alcohol and other drugs) and “Self-blame and denial” might reflect coping strategies that are frequently used by PLWH when they know about their HIV status [61, 62]. Because some PLWH would seek help from their family members, relatives, or close friends to alleviate their stress [63], the factor “Support seeking” was also identified in the study conducted by Carver et al. [20, 21]. Furthermore, some PLWH believe that spirituality would improve disease outcomes [62]; we identified the factor of “Reliance on spirituality,” which is also consistent with the emergence of religiosity in China [64].

Corroborating with previous studies that found social support predictive of problem-focused coping [37] and adaptive coping strategies [65], our study showed that social support was positively correlated with the “Problem-solving and acceptance” and “Humor and self-distraction” factors. The study also showed that “Support seeking” correlated negatively with perceived discrimination. Discrimination against PLWH exists in health service settings, and includes refusal to provide treatment and involuntary disclosure of patients’ HIV status [66–69]. Some PLWH avoid using AIDS-related services in order to protect themselves and their families from stigmatization and discrimination [38, 39]. In this study, “Support seeking” also correlated positively with perceived social support. The aforementioned findings were consistent with those from previous studies targeting Chinese PLWH [66, 70]. The results therefore provide some preliminary support to the new factor structure established by the EFA.

This study had several limitations. First, the factors derived from the EFA were sample dependent and were not cross-validated by an independent sample. We did not perform split-sample analysis including CFA for the new factor structure due to the relatively small sample size. This new factor structure is thus not conclusive and requires further validation before we can accept it. Second, the sampling frame only covered a proportion of the PLWH in the two cities (e.g., those with valid telephone numbers or those who used particular services); the sample may not be representative of the PLWH populations in the two cities although the response rate was quite high. Generalization of the results to other PLWH populations in China needs be made with caution. Third, the sample consisted of different types of PLWH, which may be seen as both a strength and a limitation. Fourth, reporting bias due to self-reporting may exist. Face-to-face interviews are, however, very common in HIV studies [71]. Literacy should not be a big problem as the majority of the sampled participants attended junior high school at the very least. Anonymous self-administered surveys could be performed. Future studies may test potential differences due to different modes of surveys, such as face-to-face interviews *versus* self-administration. Sensitive questions were asked and such topics have been covered in many PLWH studies. As the participants were interviewed by experienced service providers who knew about their HIV status and in a context of professional care, we believe that most of the participants were willing to disclose true answers. Fifth, there are some issues regarding the new factor structure. For instance, one item was double loaded on two factors and assigned to one of the two scales for clearer meaning. Furthermore, the subscale “Reliance on spirituality” only consisted of two items, which may not be satisfactory [72], although the original Brief Cope consists of 14 two-item subscales. Sixth, no additional instrument measuring coping strategies was used for testing concurrent validity of the Brief COPE, due to the length of the questionnaire. Also, the effect size of some of the significant correlations between the subscales and other measures was quite small. Seventh, this study was conducted between 2007 and 2008, but certain stressors have changed among PLWH in recent years due to improved treatment availability. However, many stressors faced by PLWH (e.g., stigma, physical problems, financial problems) and public stigma remained similar. Meanwhile, the original Brief COPE was developed in 1997 and is still widely used across study populations. We contend that the structure of coping among PLWH should be more or less stable over time. Therefore, scales developed around 2008 should still be useful for future studies on coping mechanisms among PLWH in China. Eighth, data were not collected from MMT and HIV clinics of Shenzhen city at the time of the study due to some availability and overlap issues that would produce a bias to the study results.

## Conclusions

The two original proposed factor structures of the Chinese version of the Brief COPE instrument, which has been used in a number of studies, were not found to be valid for PLWH in China. The results urge researchers to pay attention to cultural diversity when performing coping research targeting PLWH and selecting a tool to assess coping strategies. We reported a new six-factor structure established by the EFA. It represents a preliminary exploration of the factor structure of the Brief COPE instrument for the population of PLWH in China. Reassessment of the psychometric evidences of this new factor structure in PLWH and other Chinese populations is greatly warranted.

## Additional file

Additional file 1:
**Multilingual abstracts in the six official working languages of the United Nations.** (PDF 204 kb)

## Abbreviations

CDC: Center for disease control and prevention
CFA: Confirmatory factor analysis
CFI: Comparative fit index
EFA: Exploratory factor analysis
GIF: Goodness of fit index
KMO: Kaiser-Meyer-Olkin
MMT: Methadone maintenance treatment
NFI: Normed fit index
NNFI: Non-normed fit index
PLWH: People living with HIV
PDSHIV: Perceived Discrimination Scale for PLWH
PSSS: Perceived Social Support Scale
RMSEA: Root mean square error of approximation
