# Supplementary material for: A preliminary validation of the Brief COPE instrument for assessing coping strategies among people living with HIV in China
Source: Infect Dis Poverty. 2015 Sep 14;4:41. doi: 10.1186/s40249-015-0074-9 (PMC4570223; doi:10.1186/s40249-015-0074-9)
Supplement: Additional file 1: — Multilingual abstracts in the six official working languages of the United Nations. (PDF 204 kb) [file 40249_2015_74_MOESM1_ESM.pdf]

Translation of the abstract into the six official working languages of the United Nations

**التحقق المبدئي من صحة أداة COPE الموجزة من أجل تقييم استراتيجيات التعامل بين من يتعايشون مع فيروس HIV في الصين**

زياو –يو سو، جوزيف تي أف ليو، ويني دبليو ماك، كيه سي تشاو، تي – جين فينج، أكسي تشان، تشو – لاين ليو، جون ليو، دو ليو، لين تشان، جون – مين سونج، يان زيانج، جوانج –لو زيو، زيانج – ينتج زو، جين- كوان تشانج

ملخص

خلفية: لقد تم استخدام أداة COPE الموجزة لإجراء الأبحاث على العديد من السكان، بما في ذلك الأشخاص المتعايشيون مع فيروس HIV (PLWH). ولكن، عندما يتم تطبيق الاستبيان على الأشخاص المتعايشيون مع فيروس HIV (PLWH) لم يكن خاضعًا لعامل التحقق من الصحة بصورة كاملة.

الطرق: قمنا بتعيين إجمالي عدد 258 من الأشخاص المتعايشيون مع فيروس HIV (PLWH) من مقاطعتين في الصين. وقد قاموا بالإجابة على الأسئلة التي تضم مقاييس من ثلاثة أدوات: COPE الموجزة، مقياس الدعم المجتمعي المُتلقى ومقياس التفرقة الخاصة بـالأشخاص المتعايشيون مع فيروس HIV (PLWH). تم إجراء تحليل العامل التأكيدي (CFA) وتحليل العامل التفسيري (EFA).

النتائج: لقد وجد تحليل العامل التأكيدي (CFA) عدم وجود جودة الملائمة للبيانات. وقد تعرف تحليل العامل التفسيري (EFA) التالي على ستة عوامل مبدئية، تُشَكِل التراكمات بألفبائية كرونباتش، والتي تبدأ من 0.61 إلى 0.80. تم الإبلاغ عن معاملات ارتباط هامة بين التراكمات والتدابير الخاصة بالدعم الاجتماعي المُتلقى والتفرقة المتلقاة، مما يعطي الدعم المبدئي للتحقق من صحة هيكل العامل التجريبي الجديد.

الاستنتاج: أظهرت هذه الدراسة أن هيكل العامل الأصلي لأداة COPE الموجزة، عند تطبيقها على الأشخاص المتعايشيون مع فيروس HIV (PLWH) في الصين، لا يتلائم مع البيانات. وهكذا، يجب أن يتم تطبيق أداة COPE الموجزة على مختلف المجموعات السكانية والثقافية بصورة حذرةز إن هيكل العامل الجديد الذي أسسه تحليل العامل التفسيري (EFA) هو مبدئي فقط ويتطلب المزيد من التحقق من صحته.

Translated from English version into Arabic by Mohamed R. Habib, through

TRANSLATORS
WITHOUT BORDERS

**在中国艾滋病感染者中对简易应对量表（Brief COPE）进行初步效度检测**

苏小游，刘德辉，麦颖思，KC Choi, 冯铁建，陈曦，刘初良，刘军，刘德，陈琳，宋俊敏，张燕，赵广路，朱章平，程锦泉

**摘要**

**研究背景:** 简易应对量表（Brief COPE）曾用于对不同人群的应对策略的研究，包括艾滋病感染者。但之前用于艾滋病感染者研究时并未对量表进行较系统的效度检测。

**方法:**本研究招募了中国两个省份共 258 名艾滋病感染者作为研究对象。研究对象填写了 Brief COPE、感知社会支持及艾滋病感染者感知歧视问卷。统计方法采用了验证性因素分析（CFA）和探索性因素分析(EFA)。

**结果:** CFA 发现 Brief COPE 原分量表结构对数据的拟合优度不够理想，之后 EFA 产生 6 个分量表，Cronbach’s alpha 为 0.61 至 0.80。分量表同感知社会支持及感知歧视有显著相关，初步证明了 EFA 产生的 Brief COPE 量表结构的效度。

**讨论:**本研究表明原 Brief COPE 的分量表结构不适用于中国的艾滋病感染者。因此，对不同人群和不同文化背景的研究对象进行研究时，应谨慎使用 Brief COPE 量表。本研究通过 EFA 产生的分量表结构仅为初步结果，尚需进一步验证。

Translated from English version into Chinese by Su Xiaoyou.

**Validation préliminaire de l'instrument Brief COPE pour évaluer les stratégies d'adaptation parmi les personnes vivant avec le HIV en Chine**

Xiao-you Su, Joseph TF Lau, Winnie WS Mak, KC Choi, Tie-jian Feng, Xi Chen, Chu-liang Liu, Jun Liu, De Liu, Lin Chen, Jun-min Song, Yan Zhang, Guang-lu Zhao, Zhang-ping Zhu, Jin-quan Cheng

**Résumé**

**Contexte:** L'instrument Brief COPE a été utilisé pour mener une recherche sur différentes populations, y compris celles qui vivent avec le HIV, ou « PLWH » (People Living With HIV). Cependant, les aspects du questionnaire, lorsqu'ils ont été appliqués aux PLWH, n'ont pas été soumis à une validation factorielle approfondie.

**Méthodes:** Au total, 258 PLWH ont été recrutés dans deux provinces de Chine. Ils ont répondu à des questions concernant les échelles de trois instruments: le Brief COPE, l'Echelle de Soutien Social Perçu, et l'Echelle de Discrimination Perçue envers les PLWH. Une Analyse factorielle confirmatoire (AFC) et une Analyse factorielle explicative (AFE) ont été effectuées.

**Résultats:** l'AFC a trouvé une faible validité d'ajustement des données. L'AFE ultérieure a identifié six facteurs préliminaires, formant des sous-échelles avec un coefficient alpha de Cronbach allant de 0,61 to 0,80. Des coefficients de corrélation significatifs entre les sous-échelles et les mesures de soutien social perçu et de discrimination perçue ont été indiqués, donnant un soutien préliminaire au bien-fondé de la nouvelle structure factorielle empirique.

**Conclusion:** Cette étude a montré que la structure factorielle initiale de l'instrument Brief COPE, lorsqu'elle est appliquée aux PLWH en Chine, ne cadre pas avec les données. Donc, le Brief COPE devra être appliqué avec prudence aux diverses populations et cultures. La nouvelle structure factorielle établie par l'EFA n'est que préliminaire et requiert une validation ultérieure.

Translated from English version into French by Ode Laforge, through

TRANSLATORS
WITHOUT BORDERS

**Предварительная валидация инструмента Brief COPE, используемого для оценки совладающей стратегии среди людей, живущих с ВИЧ в Китае**

Сяо-юй Су, Джозеф ТФ Лю, Винни УС Мак, КС Чхве, Ци-цзянь Фэн, Си Чен, Чу-лянь Лю, Дзюн Лю, Дэ Лю, Лин Чен, Дзюн-мин Сун, Янь Чжан, Гуан-лю Чжао, Чжан-пин Чжу, Цзинь-цюань Чен

**Резюме**

**Базовая проблематика:** Инструмент Brief COPE используется для проведения исследований среди различных групп населения, включая людей, живущих с ВИЧ (ЛЖВ). Тем не менее, в случае применения к

ЛЖВ, модели вопросника до сих пор не прошли подробную валидацию факторов.
**Методы:** К участию в исследовании были привлечены 258 ЛЖВ в двух провинциях Китая. Они ответили на вопросы, связанные со шкалами трех инструментов: Brief COPE, Шкала ощущаемой социальной поддержки и шкала ощущаемой дискриминации ЛЖВ. Были проведены подтверждающий факторный анализ (ПФА) и разведочный факторный анализ (РФА).
**Результаты:** ПФА обнаружил низкую адекватность выборки. Последующий РФА идентифицировал шесть предварительных факторов, сформировав подшкалы по коэффициенту надежности Кронбаха в диапазоне от 0,61 до 0,80. Были отмечены значимые коэффициенты корреляции между подшкалами и показателями ощущаемой социальной поддержки и ощущаемой дискриминации, что является предварительным подтверждением валидности новой структуры эмпирических факторов.
**Заключение:** Данное исследование показало, что первоначальная структура факторов инструмента Brief COPE не адекватна выборке в случае его применения к ЛЖВ в Китае. Таким образом, следует с осторожностью применять Brief COPE к различным группам населения и культурам. Новая структура факторов, предложенная РФА, является предварительной и требует дополнительной валидации.

Translated from English version into Russian by Nurangiz Khodzharova, through

TRANSLATORS WITHOUT BORDERS

**Validación preliminar del instrumento Brief COPE para la evaluación de estrategias de afrontamiento en personas que viven con VIH en China.**

Xiao-you Su, Joseph TF Lau, Winnie WS Mak, KC Choi, Tie-jian Feng, Xi Chen, Chu-liang Liu, Jun Liu, De Liu, Lin Chen, Jun-min Song, Yan Zhang, Guang-lu Zhao, Zhang-ping Zhu, Jin-quan Cheng

**Resumen**
**Antecedentes:** El instrumento Brief COPE ha sido utilizado para conducir investigaciones en varias poblaciones, incluida la de personas que viven con VIH (PLWH). Sin embargo, los constructos del cuestionario, no han sido validados en profundidad cuando se aplican a PLWH.
**Métodos:** Se reclutaron un total de 258 PLWH de dos provincias de China. Dichas personas contestaron preguntas que involucraban las escalas de tres instrumentos: el Brief COPE, la escala de apoyo social percibido y la escala de discriminación percibida para PLWH. Se condujeron un análisis factorial confirmatorio (AFC) y un análisis factorial exploratorio (AFE).
**Resultados:** El AFC encontró escasa bondad de ajuste de la información. Un AFE subsecuente identificó seis factores preliminares, que formaban subescalas con los alfas de Cronbach, que variaron entre 0,61 y 8,80. Se reportaron coeficientes de correlación significativos entre las subescalas y las mediciones de apoyo social percibido y discriminación percibida, proporcionado un apoyo preliminar a la validez de la nueva estructura de factor empírica.
**Conclusión:** Este estudio mostró que la estructura original de factor del instrumento Brief COPE, cuando se aplica a PLWH en China, no se ajustaba a la información. Por lo tanto, el Brief COPE debería aplicarse con cautela a las distintas poblaciones y culturas. La nueva estructura de factor establecida por el AFE es solo preliminar y requiere

de mayor validación.

Translated from English version into Spanish by Maria Alejandra Aguada, through

TRANSLATORS
WITHOUT BORDERS
